# Absence of biofilm adhesin proteins changes surface attachment and cell strategy for *Desulfovibrio vulgaris* Hildenborough

**DOI:** 10.1128/jb.00379-24

**Published:** 2024-12-31

**Authors:** C. Pete Pickens, Dongyu Wang, Chongle Pan, Kara B. De León

**Affiliations:** 1 School of Biological Sciences, University of Oklahoma 6187 , Norman, Oklahoma, USA; 2 School of Computer Science, University of Oklahoma 542269 , Norman, Oklahoma, USA; Geisel School of Medicine at Dartmouth, Hanover, New Hampshire, USA

**Keywords:** biofilms, sulfate reduction, *Desulfovibrio*, adhesins

## Abstract

Ubiquitous in nature, biofilms provide stability in a fluctuating environment and provide protection from stressors. Biofilms formed in industrial processes are exceedingly problematic and costly. While biofilms of sulfate-reducing bacteria in the environment are often beneficial because of their capacity to remove toxic metals from water, in industrial pipelines, these biofilms cause a major economic impact due to their involvement in metal and concrete corrosion. The mechanisms by which biofilms of sulfate-reducing bacteria form, however, are not well understood. Our previous work identified two proteins, named by their gene loci DVU1012 and DVU1545, as adhesins in the model sulfate-reducing bacterium, *Desulfovibrio vulgaris* Hildenborough. Both proteins are localized to the cell surface and the presence of at least one of the proteins, with either being sufficient, is necessary for biofilm formation to occur. In this study, differences in cell attachment and early biofilm formation in single deletion mutants of these adhesins were identified. Cells lacking DVU1012 had a different attachment strategy from wild-type (WT) and ΔDVU1545 cells, more often attaching as single cells than aggregates, which indicated that DVU1012 was more important for cell-to-cell attachment. ΔDVU1545 cells had increased cell attachment compared to WT cells when grown in static cultures. To date, comparisons of the *D. vulgaris* Hildenborough have been made to the large adhesion protein system in environmental pseudomonads. Yet, we and others have shown distinct mechanistic differences in the systems. We propose to name these proteins in *D. vulgaris* Hildenborough biofilm formation system to facilitate comparisons.

**IMPORTANCE:**

Biofilms of sulfate-reducing bacteria contribute to biocorrosion, costing the United States hundreds of millions of dollars annually. In contrast, these biofilms can be used to bioremediate toxic heavy metals and to generate bioelectricity. As one of the most abundant groups of organisms on Earth, it is pertinent to better understand mechanistically how the biofilms of sulfate-reducing bacteria form so we may use this knowledge to help in efforts to mitigate biocorrosion, to promote bioremediation, and to produce clean energy. This study shows that the absence of either one of two biofilm adhesins impacts surface colonization by a sulfate-reducing bacterium, and that these two biofilm adhesins differ in their effect on cell attachment compared to other well-documented bacteria such as *Pseudomonas* species.

## INTRODUCTION

Ubiquitous in nature, biofilms are densely packed microbial cells attached to surfaces that envelop themselves in a matrix of extracellular polymeric substance made of carbohydrates, proteins, DNA, and other polymers. Growth as a biofilm is evolutionarily advantageous as it gives some stability in a constantly fluctuating environment and provides protection from a variety of environmental challenges, including UV, acid, dehydration, salinity, metal toxicity, phagocytosis, and several antimicrobial agents (1). In subsurface environments, the active microbial population is predominantly found attached to surfaces as a biofilm (2).

Biofilms of sulfate-reducing bacteria (SRB) impact the world around them in multiple ways. SRB biofilms are the major contributors to microbiologically influenced corrosion, causing millions of dollars in damages annually to the United States (3). SRB biofilms can also assist with bioremediation, where SRB biofilms precipitate heavy metals such as chromium and uranium and allow for their removal from contaminated groundwater (4, 5). The environmental effects caused by SRB biofilm showcase their impact and importance in industrial and environmental systems. Biofilms of SRB have also been shown to play a role in animal gut colonization and have been shown to affect immune responses in hosts through the production of sulfide gas (6). These effects and outcomes of SRB biofilm have been well-documented, affecting multiple different environments and having potentially positive and negative effects in their immediate environment.

Biofilms formed by the model SRB, *Desulfovibrio vulgaris* Hildenborough (7), have previously been shown to be predominantly protein and are in a distinct state of growth for *D*. *vulgaris* cells compared to cells growing unassociated with a surface (8, 9). Under conditions similar to those used in the present study, biofilms of *D. vulgaris* Hildenborough have been shown to contain minimal amounts of pentose, hexose, and uronic acids, with a ratio of carbohydrate-to-protein less than that of planktonic cells, and the biofilms are susceptible to protease treatment (8). This suggests that proteins play a dominant and essential role in *D. vulgaris* Hildenborough biofilm formation. Previous work identified the importance of a type I secretion system and two biofilm adhesins in biofilm formation under continuous culture and flow (10). Those proteins are annotated as hemolysin-like calcium-binding proteins and are called by their gene loci, DVU1012 and DVU1545. The absence of either one of these two proteins caused no difference in biofilm formation over time compared to the wild-type (WT) strain. However, in the absence of both proteins, no biofilm was formed.

There are other examples of biofilm adhesins affecting surface colonization and biofilm maturation across other bacterial species. In both *Pseudomonas fluorescens* and *P. putida*, the proteins large adhesion proteins LapA and LapF share similarities to *D. vulgaris* proteins DVU1012 and DVU1545, respectively (11, 12). Both proteins have been well-characterized for their role in surface colonization and biotic attachment. LapA and LapF also utilize a type I secretion system (LapBC and TolC) for export out of the cell, and both are retained in the outer membrane. However, mutants lacking the protein LapA were found to be deficient in surface colonization and whole biofilm formation over time compared to LapF, whereas the protein LapF was found to have a greater impact on cell-to-cell adhesion and later-stage biofilm development (11, 12). Another protein in *P. fluorescens* Pf0-1, MapA, also shares similarities with LapA; both utilize a type I secretion system, the retention is regulated by intracellular levels of cyclic-di-GMP, and both affect biofilm formation by the host bacterium (13). While LapA, LapF, and MapA each have their own type I secretion systems encoded adjacent to their respective genes, there is evidence that MapA is moderately exported by the LapBCE secretion system of LapA when its cognate system, MapBCE, is absent (12). To our knowledge, LapF has not been shown to be exported by a different type I secretion system than its cognate system. MapA has also shown differential expression to LapA within biofilms, with expression of MapA primarily within maturing biofilms compared to the constitutive expression of LapA. These proteins have served as examples of biofilm formation for homolog adhesins across other species and have shown how multiple biofilm adhesins within a host cell can perform different functions.

For *D. vulgaris* Hildenborough biofilm proteins DVU1012 and DVU1545, their distinct functional roles have not yet been determined. Both DVU1012 (3038 AA) and DVU1545 (2414 AA) proteins are mostly comprised of different low-complexity repeats with a calcium-binding type I secretion recognition motif at their C-termini. DVU1012 also has a von Willebrand type A domain located between the repeats and the recognition motif. The protein DVU1012 was previously found to be the most abundant protein within the extracellular matrices of the biofilm and has been shown to have high abundance within planktonic cultures as well (9, 14). The other biofilm-associated protein DVU1545, however, is found in lower abundance compared to DVU1012. Since the absence of either DVU1012 or DVU1545 does not impact biofilm formation, biofilms formed by *D. vulgaris* must then compensate for the absence of one of the most abundant proteins within the extracellular biofilm matrix. Previous studies have identified that both proteins are exported out of the same type I secretion system in *D. vulgaris* Hildenborough, both proteins are large, and both proteins are localized to the surface after export (10). Also, the absence of DVU1012 does not influence biofilm maturation over time, unlike the mutation of the analogous protein LapA in *P. fluorescens* and *P. putida* (11). Retention of the protein LapA on the surface of the cell is determined by the activity of the periplasmic protease LapG, which in turn is regulated by the c-di-GMP sensing protein LapD; no such retention regulation mechanism has been shown for the protein LapF. DVU1019 and DVU1020 in *D. vulgaris* Hildenborough have been shown to perform similar functions to LapG and LapD, respectively (15). Recently, the retention module portion of the protein DVU1012, when heterologously expressed in *P. fluorescens* Pf0-1, was found to be acted upon by DVU1019 and DVU1020 in a c-di-GMP-dependent manner (15). Another group had previously reported the role of c-di-GMP in modulating biofilm formation in *D. vulgaris* Hildenborough through the screening of transposon mutants (16); however, the transposon mutant library was constructed in the Missouri or “MO” parental strain subsequently found to be deficient in biofilm formation (10).

As the proteins LapA and LapF have been shown to have distinct roles for biofilm growth in environmental *Pseudomonas* species, the proteins DVU1012 and DVU1545 may also play differing roles in surface colonization and maturation. While the consequences and outcomes of SRB biofilms are well understood, the mechanisms used by SRB to colonize surfaces, co-aggregate, and form biofilm are not. As complete redundancy of proteins is rare in microbes (17), we hypothesize that the two proteins DVU1012 and DVU1545 have distinct, but overlapping roles in cell attachment and biofilm formation. In this study, proteomics was used to assess whether the absence of either biofilm adhesin caused a proportional change in the other adhesin or in any other proteins within the biofilm matrix. Early static biofilms were quantified to determine differences in biofilm maturation at different development stages and under differing environmental conditions. Surface colonization and cell aggregation of *D. vulgaris* adhesin protein mutants were assessed using real-time phase contrast microscopy. This study highlights the effects of the biofilm adhesin deletion on mature biofilms of *D. vulgaris* Hildenborough and the major differences between the functional roles of SRB adhesins DVU1012 and DVU1545 compared to LapA and LapF in the environmental pseudomonads.

## RESULTS

### Abundance of adhesin proteins DVU1012 or DVU1545 does not change in mutant biofilms lacking the other adhesion

**Fig 1:**
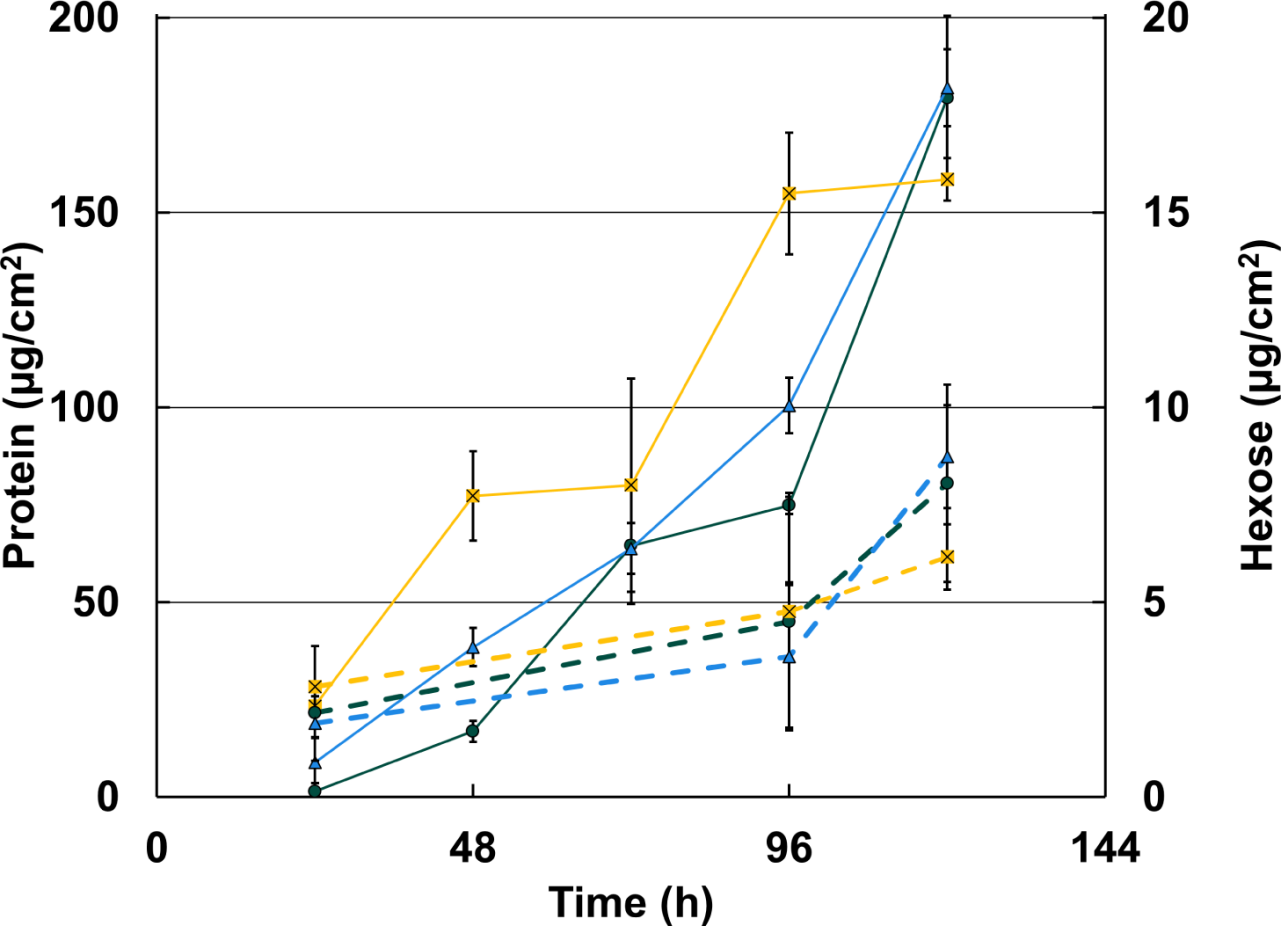
Biofilm maturation over time for WT and the biofilm adhesin mutants. Protein (solid lines) and hexose (dashed lines) as micrograms per square centimeter over time for WT (dark teal, circle), ΔDVU1545 (yellow, square), and ΔDVU1012 (blue, triangle). Error bars indicate standard deviation across technical replicates (*n* = 3).

**Fig 2:**
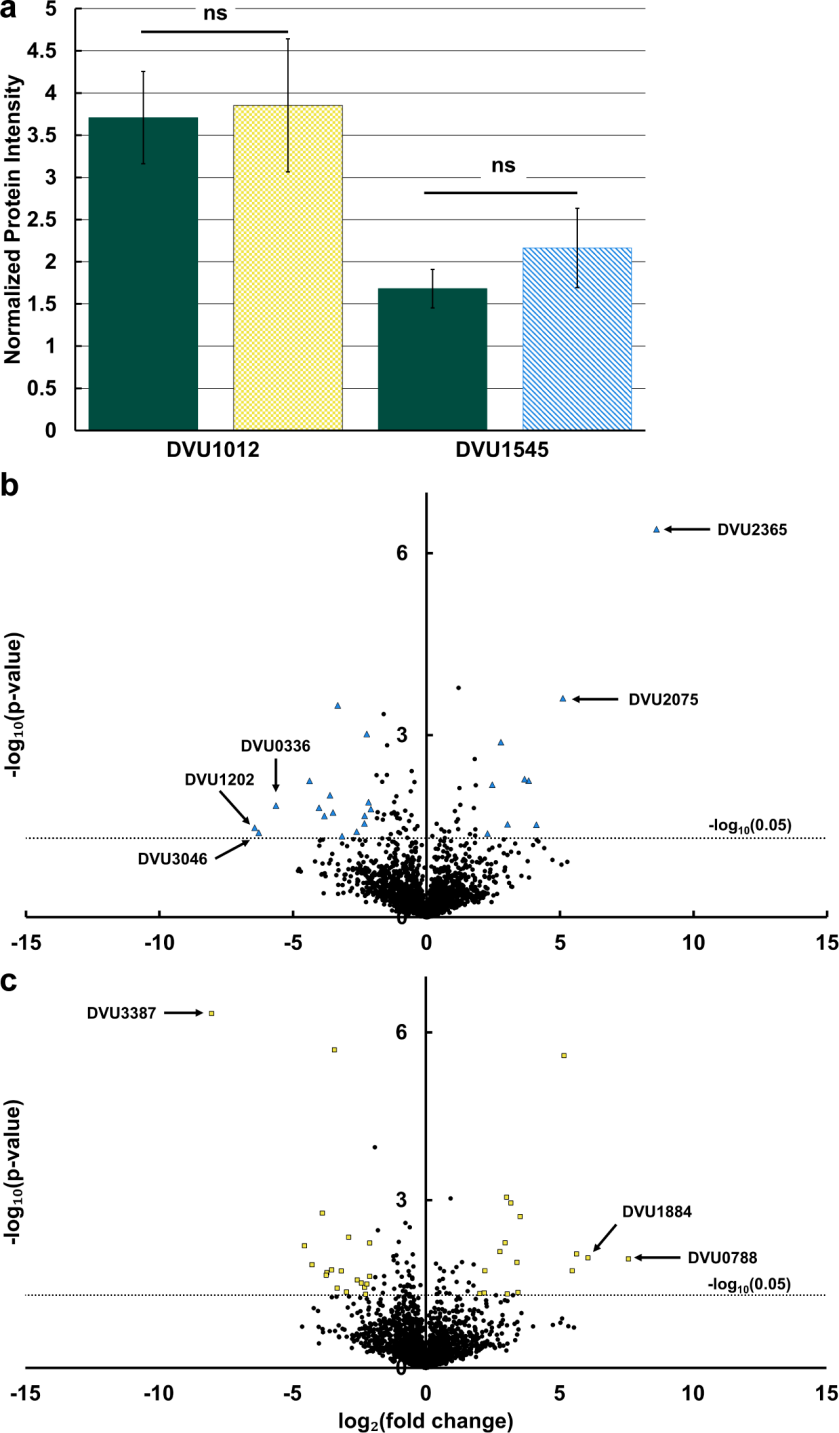
Changes in protein abundance between biofilms of WT and the biofilm adhesin mutants. (**A**) Normalized protein intensity for DVU1012 and DVU1545 for WT (dark teal, solid), ΔDVU1545 (yellow, checker), and ΔDVU1012 (blue, slant). Error bars indicate standard deviation across replicates (*n* = 3). ns, non-significant differences as determined by a homoscedastic Student’s *t* test (*P* > 0.05). The relative fold change in abundance of proteins were compared between WT and ΔDVU1012 (**B**) and WT and ΔDVU1545 (**C**). Dotted line denotes the cutoff for statistical significance (*P* < 0.05). Data points with larger than a fourfold change in abundance and statistically significant are marked for ΔDVU1012 (blue, triangle) and ΔDVU1545 (yellow, square). The gene loci of selected proteins are labeled with arrows. The complete lists of significant proteins are shown in Tables 1 and 2.

Monitoring biofilm growth on glass over time, no significant differences in total biomass were measured between the single deletion strains of DVU1012*,* DVU1545*,* and the parental strain, with minimal differences in measured proteins or hexose per square centimeter (Fig. 1). This corresponds with previous findings with protein measurements from *D. vulgaris* Hildenborough biofilms (10). Carbohydrate measurements for biofilms of the single deletion mutant strains have not previously been published, and no significant differences were found between the strains. This general trend was also found for biofilm growth on mild steel (Fig. S1). The single mutants formed biofilm on mild steel indicating either protein is sufficient for biofilm formation, though the strain lacking the protein DVU1545 did have less biofilm formation than the parental strain and the strain lacking DVU1012. The double mutant had detectable protein on the steel surface, but it did not accumulate over time. Since there was no difference in measured biofilm protein between the strains and the biofilm matrices were still predominantly protein, proteomic analyses were performed to determine differences in biofilm protein composition. Whole biofilm samples from 72 h for each strain were used in proteomic analyses (Table S1). Comparing normalized protein intensities, which are on a log_2_-based scale, DVU1012 was approximately fourfold more abundant than DVU1545 in WT cells (Fig. 2A). Overall, the absence of either biofilm adhesin does change the biofilm protein compositions between the single deletion mutants and WT cultures (Fig. 2B and C). Interestingly, when comparing the relative protein abundance of the proteins DVU1012 and DVU1545 between WT and the single deletion mutants, the abundance of the proteins DVU1012 and DVU1545 was not different between WT and the corresponding single deletion mutants (Fig. 2A). In addition, few other proteins changed in abundance between WT and the single deletion mutants (Fig. 2B through C). Biofilms formed by the ΔDVU1012 strain had a decreased abundance of proteins related to cell wall/membrane/envelope biogenesis and coenzyme transport and metabolism (Table 1). Most proteins with different abundance in biofilms of the ΔDVU1012 strain when compared to WT were predicted to be localized to the cytoplasm, and none of the proteins were predicted to be localized to the inner or outer membrane. This differs when looking at the proteins with different abundances in ΔDVU1545 biofilms compared to WT, as four proteins are predicted to localize to the inner membrane (Table 2). Proteins related to cell wall/membrane/envelope biogenesis were increased overall in ΔDVU1545 biofilms compared to WT, and proteins predicted to be involved in coenzyme transport and metabolism were decreased. None of the proteins with different abundances in the single deletion strains compared to WT had annotations that supported a role in biofilm matrix formation. Thus, overall, the absence of either biofilm adhesin protein did not cause a significant change in the abundance of the other protein, and another protein did not apparently compensate for the missing adhesin.

**TABLE 1:** Proteins with significant fold change differences between ΔDVU1012 and WT

Gene locus ID	Description	Functional code^*a*^	Predicted localization^*b*^	Fold change	*P* value
DVU2365	Conserved hypothetical protein	S	Cytoplasm	8.615	4.01E−07
DVU2075	ParA family protein	D	Cytoplasm	5.104	2.47E−04
DVU2135	Hypothetical protein		Extracellular	4.114	0.030
DVU3165	Conserved domain protein		Cytoplasm	3.822	0.006
DVU0573	CreA protein	S	Unknown	3.667	0.005
DVU2057	Conserved hypothetical protein	S	Cytoplasm	3.031	0.029
DVU0959	Replicative DNA helicase	L	Cytoplasm	2.787	0.001
DVU2577	DNA-binding response regulator, LuxR family	TK	Cytoplasm	2.463	0.007
DVU1980	Hypothetical protein		Unknown	2.288	0.042
DVU1200	Riboflavin synthase, alpha subunit	H	Cytoplasm	−2.088	0.017
DVU0984	tRNA-i (6)A37 modification enzyme MiaB	J	Cytoplasm	−2.169	0.013
DVUA0019	Type II DNA modification methyltransferase, putative		Cytoplasm	−2.239	0.001
DVU0829	Phosphoenolpyruvate-protein phosphotransferase	G	Cytoplasm	−2.316	0.021
DVU0469	N-(5-phosphoribosyl)anthranilate isomerase	E	Cytoplasm	−2.325	0.028
DVU1420	Hpt domain protein	T	Unknown	−2.619	0.039
DVU3227	Flagellar basal body-associated protein, putative	N	Unknown	−3.163	0.046
DVU1596	Protein-glutamate methylesterase CheB	NT	Cytoplasm	−3.326	3.22E−04
DVU0282	A/G-specific adenine glycosylase	L	Cytoplasm	−3.501	0.019
DVU1365	Heme-binding protein, putative	H	Unknown	−3.612	0.010
DVU2748	Precorrin-4 C11-methyltransferase	H	Cytoplasm	−3.823	0.021
DVU0135	Conserved hypothetical protein	D	Cytoplasm	−4.027	0.016
DVU3265	Tartrate dehydratase beta subunit, putative	C	Cytoplasm	−4.379	0.006
DVU0336	Aminotransferase, DegT/DnrJ/EryC1/StrS family	M	Cytoplasm	−5.638	0.014
DVU3046	Glycosyl transferase, group 1 family protein	M	Cytoplasm	−6.283	0.040
DVU1202	Cytidine/deoxycytidylate deaminase family protein	F	Unknown	−6.439	0.033

^*a*^ Functional codes based on Clusters of Orthologous Groups (COGs).

^*b*^ Localization of proteins predicted by PSORTb.

**TABLE 2:** Proteins with significant fold change differences between ΔDVU1545 and WT

Gene locus ID	Description	Functional code^*a*^	Predicted Localization^*b*^	Fold change	*P* value
DVU0788	Rod shape-determining protein MreC	M	Unknown	7.584	0.011
DVU1884	Methyl-accepting chemotaxis protein	NT	Inner membrane	6.066	0.011
DVU1165	NADH respiratory dehydrogenase	C	Inner membrane	5.639	0.009
DVU2057	Conserved hypothetical protein	S	Cytoplasm	5.481	0.018
DVU2859	Tail sheath protein, putative	R	Unknown	5.177	2.59E−06
DVU3165	Conserved domain protein		Cytoplasm	3.529	0.002
DVU0888	Response regulator	T	Cytoplasm	3.449	0.045
DVU0867	Aromatic amino acid decarboxylase, putative	E	Cytoplasm	3.406	0.013
DVU0959	Replicative DNA helicase	L	Cytoplasm	3.187	0.001
DVU1889	Phosphoheptose isomerase	G	Cytoplasm	3.048	0.048
DVU0573	CreA protein	S	Unknown	3.022	0.001
DVU1651	Conserved hypothetical protein		Inner membrane	2.964	0.006
DVU2063	Conserved hypothetical protein	L	Cytoplasm	2.773	0.008
DORF41366	Hypothetical protein		Unknown	2.204	0.018
DVU1360	UDP-glucose 4-epimerase	M	Cytoplasm	2.186	0.045
DVU2512	S-adenosyl-methyltransferase MraW	M	Cytoplasm	2.013	0.047
DVU2070	TPR domain protein	S	Unknown	−2.102	0.023
DVU3223	Aspartate aminotransferase	E	Cytoplasm	−2.107	0.006
DVU0091	Conserved hypothetical protein		Unknown	−2.211	0.032
DVU0606	Transcriptional regulator, ArsR family/methyltransferase, UbiE/COQ5 family	H	Cytoplasm	−2.256	0.048
DVU1346	Exodeoxyribonuclease VII, large subunit	L	Cytoplasm	−2.300	0.036
DVU0401	Hypothetical protein		Unknown	−2.421	0.030
DVU0659	Hypothetical protein		Unknown	−2.567	0.027
DVU3310	ATP-dependent RNA helicase, DEAD/DEAH family	LKJ	Cytoplasm	−2.895	0.005
DVU1423	2-oxoglutarate dehydrogenase, E3 component, lipoamide dehydrogenase	C	Cytoplasm	−2.970	0.044
DVU1775	3,4-dihydroxy-2-butanone 4-phosphate synthase	H	Cytoplasm	−3.165	0.018
DVU0336	Aminotransferase, DegT/DnrJ/EryC1/StrS family	M	Cytoplasm	−3.318	0.037
DVU0821	Conserved hypothetical protein		Unknown	−3.428	2.04E-06
DVU2062	ATP-dependent DNA helicase, UvrD/REP family	L	Cytoplasm	−3.529	0.018
DVU1559	Aldehyde oxidoreductase	C	Cytoplasm	−3.708	0.020
DVU2655	d-alanyl-d-alanine carboxypeptidase family protein	M	Inner membrane	−3.733	0.022
DVU3049	Hemerythrin family protein	P	Cytoplasm	−3.874	0.002
DVU1365	Heme-binding protein, putative	H	Unknown	−4.265	0.014
DVU3387	Hypothetical protein		Unknown	−4.544	0.007

^*a*^ Functional codes based on Clusters of Orthologous Groups (COGs).

^*b*^ Localization of proteins predicted by PSORTb.

### Absence of protein DVU1545 increases biofilm formation under batch culture and static conditions

**Fig 3:**
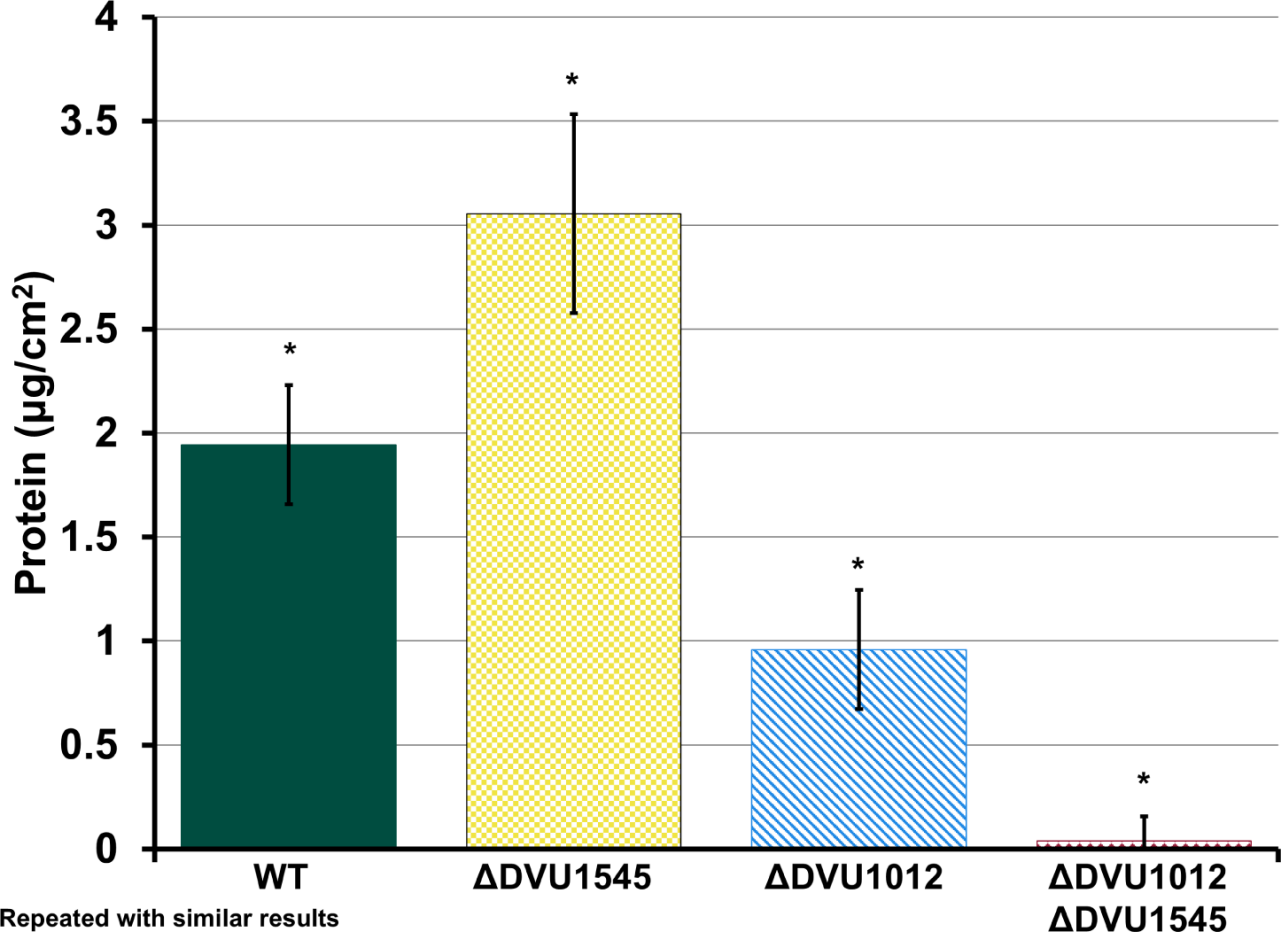
Early biofilm growth under static conditions for wild-type and the biofilm adhesin mutants. Protein (µg/cm^2^) of biofilms formed by WT and the biofilm adhesin deletion mutants. Error bars indicate standard deviation across replicates (*n* = 3). Asterisks above data points denote statistical significance as determined by a one-way ANOVA and Tukey Kramer *post hoc* test (*P* < 0.01).

Our previous experiments comparing whole biofilm growth over time of WT and single deletion mutants in CDC bioreactors did not begin measurements of biofilm biomass until 48 h post-inoculation, following 24 h of batch growth and 24 h of continuous flow (10). At this time point, the ΔDVU1545 strain consistently had more biofilm biomass than WT and the biofilm of ΔDVU1012 strain was often not detectable. Yet, all strains formed similar amounts of biofilm during maturation. To determine whether the absence of either one or both biofilm adhesins affected early biofilm growth; biofilm samples from WT, single deletion mutants, and the double deletion mutant cultured under batch, static conditions within Balch tubes were compared after 24 h of growth (Fig. 3). There were no significant differences in growth rate between the strains (growth rate constants; h^−1^ ± standard deviation: WT, 0.16 ± 0.01; ΔDVU1545, 0.17 ± 0.01; ΔDVU1012, 0.17 ± 0.01; and ΔDVU1012 ΔDVU1545, 0.16 ± 0.01), and so differences in biofilm biomass would not be from differences in growth rate but due to the effect of the deletion of DVU1012 and/or DVU1545. Biofilms were grown statically in batch cultures for 24 h (approximately six doublings) as this allowed for biofilm biomass to be quantifiable, as 16 h was the earliest that biofilm biomass could reliably be measured by protein in the WT cultures. At 24 h, the cells would be in log phase and, thus, not considered to be nutrient limited under these culturing conditions. The ΔDVU1545 strain had increased biofilm compared to all other strains (Fig. 3). The ΔDVU1012 strain had less biofilm compared to both WT and ΔDVU1545. The double deletion mutant had little to no biofilm formation, consistent with our previous findings from culturing biofilms under continuous culture and shear conditions (10).

*D. vulgaris* strain DP4 (formerly strain DePue) is highly similar to *D. vulgaris* Hildenborough, having an average nucleotide identity of 98.77% (18, 19), but a homolog for DVU1545 is missing in this strain. The same experiment for biofilm formation under static culture conditions was performed with WT, ΔDVU1545, and strain DP4 to determine whether the closely related strain formed comparable biofilm biomass to the ΔDVU1545 strain. DP4 formed more biofilm than *D. vulgaris* Hildenborough WT but less biofilm than the ΔDVU1545 strain and was not significantly different from either (Fig. S2). Nevertheless, this supported the observation that the absence of DVU1545 may result in more biofilm biomass in early biofilms under these conditions.

### Absence of biofilm proteins DVU1012 and DVU1545 affects surface colonization and cell aggregation

**Fig 4:**
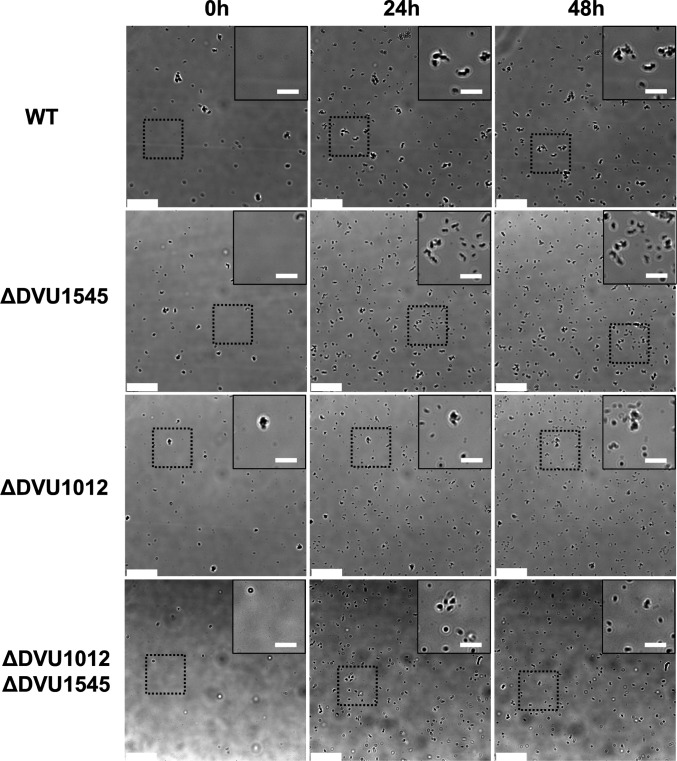
Phase contrast microscopy of cells on the glass surface. Images are shown for WT and the biofilm adhesin mutants at 0, 24, and 48 h post-inoculation. The scale bar at the bottom left of each image represents 30 µm. The inset shows an enlargement of the same region shown in the dotted square across time with a scale bar that represents 10 µm. The imaging position shifted slightly during the 48-h experiment due to the mechanical stage movement. This is visible by the slight shift in the dotted box position within the main image over time.

Real-time microscopy was used to image biofilm formation over time within recirculating capillary reactors for WT and the deletion mutants to determine differences in surface colonization between the strains. Cultures were recirculated at a rate corresponding to the relative shear force experienced when cultured within the CDC bioreactors (20). Images were taken every 10 min for 48 h, with five positions imaged in each capillary reactor across three separate capillary reactor experiments per strain. Monitoring biofilm formation in real time revealed differences in cell strategy and surface colonization between WT and the biofilm matrix protein deletion strains (Fig. 4). WT and ΔDVU1545 cultures had higher proportions of cell aggregation compared to ΔDVU1012 and the double deletion mutant cultures (Fig. 4). Cells in WT cultures are more often attached as cell aggregates rather than single cells. In contrast, the ΔDVU1012 cultures showed little aggregation, and many observed attachment events were by single cells (Fig. 4). While monitoring attached ΔDVU1012 cells over time, few microcolonies formed, though the cells appeared to be alive and were likely capable of replication.

**Fig 5:**
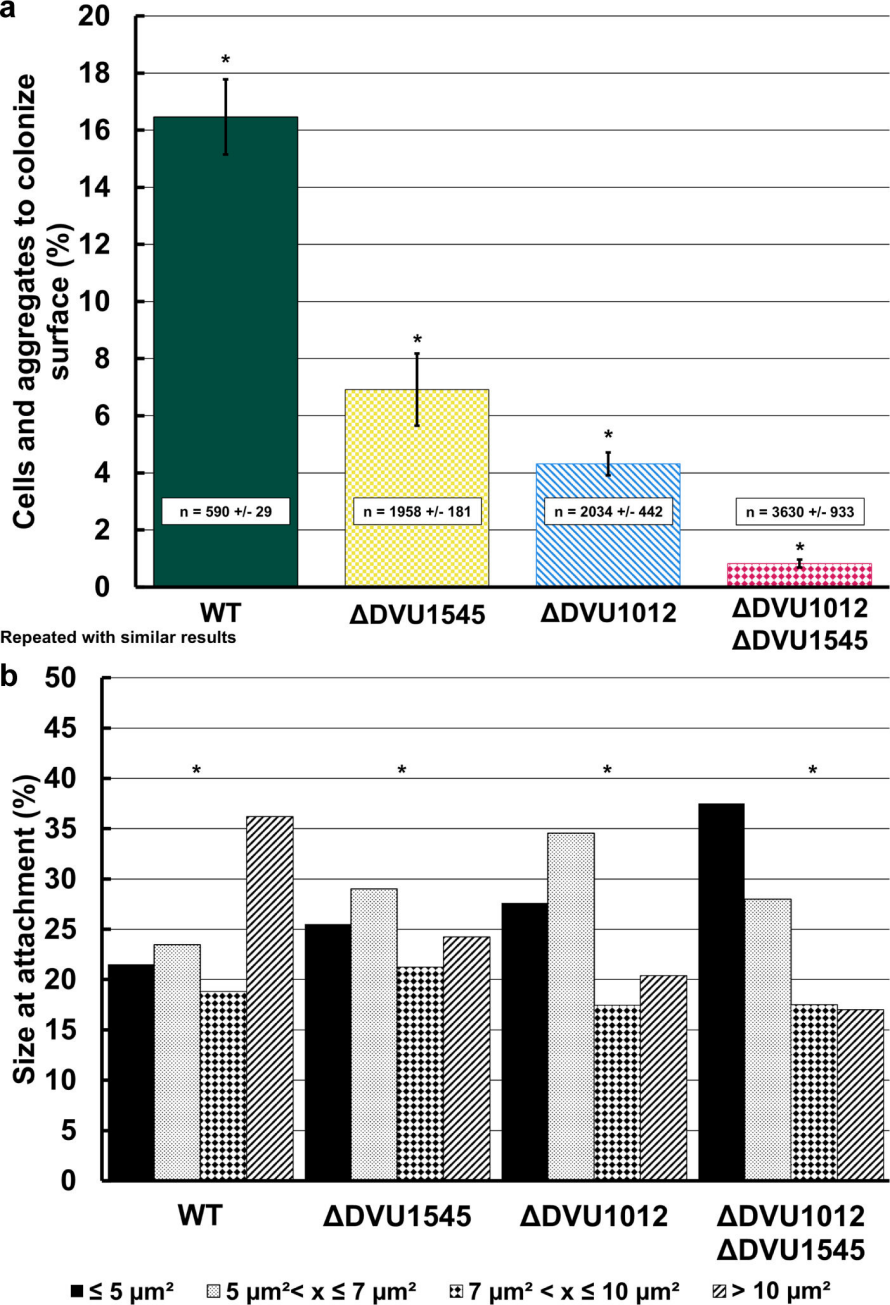
-Differences in surface attachment and cell strategy of wild-type and the biofilm adhesin mutants. (**A**) The percentage of cells and cell aggregates to become surface colonized as defined as entering the frame after the start of the experiment and remaining in the position for a minimum of 16 h. Error bars indicate standard deviation across replicate positions (*n* = 5). The asterisks indicate statistical significance from a one-way ANOVA and Tukey Kramer *post hoc* test (*P* < 0.05). The average number and standard deviation of cells and cell aggregates tracked across replicate experiments is shown in text boxes on each bar. (**B**) Size distribution of surface-attached cells and cell aggregates at initial attachment for WT and biofilm adhesin mutants. The four size ranges selected are the quartiles for the entire data set. The asterisks indicate statistical significance calculated using the Kruskal Wallis test and Conover *post hoc* test (*P* < 0.05).

By tracking cells and cell aggregates throughout the experiment, the time that they remained attached was measured to differentiate reversibly attached cells from irreversibly attached cells (those considered to be surface colonized). We defined surface colonization as cells and cell aggregates that came into frame after the start of the experiment and remained in one position for at least 16 h. This length of time was determined by comparing the percentages of surface colonization at different lengths of time in increments of 1 h. We found that after 16 h, the change in surface colonization percentages minimally changed, meaning if the cell or cell aggregate attached for 16 h, it was likely to remain attached for the duration of the experiment. So, we set the minimum time for defining colonization at this time. A significantly smaller proportion of tracked cells and cell aggregates of single deletion mutants colonized the surface compared to WT under these conditions (Fig. 5A). The absence of DVU1012 caused an even greater reduction in surface colonization than the absence of DVU1545. The double deletion mutant strain lacking both DVU1012 and DVU1545 showed minimal surface colonization. The size of cells or cell aggregates that colonized the glass surface was measured at the time of attachment by particle tracking software (Fig. 5B). WT cultures had the highest proportion of cell aggregates greater than 10 µm^2^ when first attached. The ΔDVU1012 cultures had lower proportions of attached aggregates greater than 7 µm^2^, and the double deletion mutant had few attachment events and, of those that attached, most were <5 µm^2^ and likely were single cells. Thus, surface colonization was impacted by the loss of either DVU1012 or DVU1545 and DVU1012 was important for cell aggregation and microcolony formation.

## DISCUSSION

We previously identified two proteins in the sulfate-reducing bacterium *D. vulgaris* Hildenborough, DVU1012 and DVU1545, localized to the cell surface by a type I secretion system and with similar sequence motifs to LapA and LapF, respectively (10). However, our single deletion mutants of DVU1012 or DVU1545 did not have a loss in the biofilm phenotype and had similar biofilm formation rates to WT when grown on glass (10). The double deletion mutant was deficient in biofilm formation; thus, at least one of these biofilm proteins is required for biofilm protein, but either adhesin is sufficient. Localization of at least one of these proteins to the cell surface is also required for colonization of the rat colon (21). In this present study, these proteins were also found to be involved in attachment to mild steel (Fig. S1). To begin to better understand these proteins play in biofilm formation and colonization, we aimed to determine differences in biofilm protein composition between the WT and single deletion strains on glass, as well as determine the differences in cell strategy and surface colonization by WT, the single deletion mutants, and the double deletion mutant by monitoring biofilm formation in real-time.

The relative abundance of proteins DVU1012 and DVU1545 was not significantly different between WT and the single deletion mutants (Fig. 2A). The protein DVU1012 was approximately four times more abundant than DVU1545 which is similar to what has been reported previously (9, 22). The protein DVU1012 was previously found as the most abundant protein within the extracellular matrix of biofilms formed by *D. vulgaris* (9) and DVU1012 was ranked 80th in our whole-cell proteomics data and the most abundant protein predicted to be extracellular. This indicates that the absence of one biofilm protein does not lead to an increase in the other protein’s abundance, and that the proteins do not directly compensate for the other within the biofilm matrix. Yet, there is no reduction in whole biofilm biomass, measured as protein, between the strains when the gene is removed from the bacterium. Based on the proportion of the protein DVU1012 within the biofilm proteome of WT, we estimated that the protein DVU1012 abundance to be 0.14 µg/cm^2^. When comparing biofilm mass coverages of biofilms between WT and ΔDVU1012, this falls within the standard deviation of both strains. From the proteomics data, we did not determine a clear change in biofilm matrix protein composition between the strains except for the absence of the deleted adhesin in each single deletion mutant and a hypothetical protein encoded by DVU2135 that was increased in the ΔDVU1012 strains and was predicted to localize to the extracellular matrix, but its function is unknown. Many of the proteins that did significantly change in abundance were associated with cell wall structure and chemotaxis. The proteins with the largest increase in abundance within biofilms of ΔDVU1012 strain compared to WT are predicted to be involved in cell wall structure and in chemotaxis (encoded by DVU2365 and DVU2075, respectively). (Fig. 2B). Chemotaxis may play a role in the cell reaching the surface. Though a lag in cell attachment in the capillary flow cells was not observed, the ΔDVU1012 strain formed fewer aggregates and attached more often as single cells. This would suggest that a higher proportion of the proteins within the biofilm may need to express chemotaxis proteins than cells within an aggregate population. The rates of cell attachment to the surface as DVU2365, annotated as a conserved hypothetical protein, has 51% identity to the UDP-2,3-diacylglucosamine diphosphatase, LpxI, from *D. ferrophilus* IS5, which is involved in lipid biosynthesis and is increased in expression within biofilms on carbon steel surfaces (23). Interestingly, the proteins with the largest decrease in abundance within biofilms of the ΔDVU1012 strain are also predicted to be involved in the cell wall structure (e.g., DVU1202, DVU3046, and DVU0336). A similar trend occurred in biofilms of the ΔDVU1545 strain compared to WT. The proteins encoded by DVU0788 and DVU1884, a rod-shape determining protein MreC and methyl-accepting chemotaxis protein, respectively, showed the highest increase in the abundance in biofilms of the ΔDVU1545 strain and are also predicted to be involved in cell shape and chemotaxis. The chemotaxis protein encoded by DVU1884 is predicted to contain a PAS domain, which has been linked to biofilm regulation in other species such as *Pseudomonas aeruginosa* (24). The protein with the largest decrease in abundance, encoded by DVU3387, has predicted domains related to glycosyl hydrolase enzymes and thus also related to cell wall synthesis. These changes in abundance of proteins related to cell wall, cell partitioning, and chemotaxis for both single deletion mutants suggest that there are additional changes in cell surface composition in the absence of either biofilm adhesin. Both adhesion proteins are abundant within WT cells, and so their absence likely requires a change in the cell surface composition to compensate and maintain the integrity of the membrane. Thus, it is possible that the protein changes within the biofilm are a general response to maintain cell integrity rather than a biofilm-specific response. An alternative could be that these biofilm matrix proteins play a role in surface-sensing by the cell and a subsequent signal transduction to initiate a transition to irreversible attachment, and that the changes observed in the proteome were due to the loss of this signal.

While measuring biofilm formation over time under different growth conditions, the rates of biofilm maturation in the WT and single deletion mutants were similar in the CDC reactors (Fig. 1), as has been shown previously (10). However, the measurement of early biofilms cultivated under static conditions did reveal biofilm phenotypic differences between the strains. The absence of protein DVU1012 caused a significant decrease in biofilm under static conditions, whereas the inverse was seen in the absence of protein DVU1545 (Fig. 3). This increase in biofilm by the ΔDVU1545 strain was not observed under shear conditions in the CDC reactors (Fig. 1) or capillary reactors (Fig. 5). We observed more cell attachment events in capillary reactors with the ΔDVU1545 strain than the WT strain, but more of these cells were reversibly attached and did not colonize the surface. *D. vulgaris* strain DP4 is highly similar to *D. vulgaris* Hildenborough genetically, but is missing a homolog for DVU1545 (18). To our knowledge, DP4 biofilms have not been described previously. In this study, DP4 formed more biofilm under static conditions when compared to *D. vulgaris* Hildenborough WT though it was not significantly different (Fig. S2). Thus, the absence of DVU1545 in the mutant strain of *D. vulgaris* Hildenborough and a homolog in WT DP4 may impact resistance to shear and/or a transition from reversible to irreversible attachment.

Real-time monitoring of biofilm formation by the strains revealed differences in cell strategy and surface colonization by WT and biofilm matrix protein deletion mutants. The absence of either biofilm adhesin caused a decrease in surface colonization compared to WT, with a greater effect seen in the absence of DVU1012 (Fig. 5A). This indicates the loss of protein DVU1012 affects surface colonization more so than the protein DVU1545. Results from comparing the proportion of sizes of tracked objects (cells and cell aggregates) when first attached showed WT cultures had the highest proportion of cell aggregates compared to the other strains and most often attached as cell aggregates (Fig. 5B). Cells lacking DVU1545 had a more even distribution of particle sizes and were attached as both individual cells and cell aggregates. Cells lacking DVU1012 tended to attach more often as single cells, and few colonized cells formed microcolonies over time. The attached cells were likely active and capable of cell division, but the daughter cells did not remain on the surface or did not remain adjacent to the parent cell upon division. This suggests that biofilms of cells lacking DVU1012 have more confluent growth across the surface that eventually fills the surface area to then establish a mature biofilm. The formation of microbial aggregates and biofilm increases resistance and resilience to stressors such as shear, antibiotic stress, and natural surfactants (25), so the smaller and fewer cell aggregates formed by the ΔDVU1012 strain may make these cells more vulnerable to stress and strongly select for retention of this large, abundant protein that is energetically costly to maintain. Whether biofilms of these single deletion strains have different responses to stresses warrants further inquiry.

Overall, we conclude that the absence of either biofilm adhesin does not cause a proportional change in the abundance of the other biofilm adhesin, nor does it cause proportional changes in other proteins that show potential to compensate within the biofilm matrix. The absence of DVU1012 impacts cell aggregation and the absence of DVU1545 leads to phenotypic differences depending on shear conditions. This work has further illuminated the knowledge of biofilm adhesins in *Desulfovibrio* species and showed differences in biofilm adhesin functionality compared to the Lap system in *Pseudomonas* spp. We have shown physiological differences between the two proteins, DVU1012 and DVU1545, and how they differ from the proteins LapA and LapF, important for surface attachment and cell attachment, respectively, in environmental pseudomonads. Another biofilm protein in *Pseudomonas* species, MapA, shares high sequence similarity with LapA, but not LapF (13). Proteins DVU1012 and DVU1545 sequences are not similar, and their domains are like LapA and LapF, respectively (10). MapA can use the secretion system of LapA (13) like DVU1012 and DVU1545 can and is also controlled by LapG and LapD which has been shown for DVU1012 (15) and we predict is the case with DVU1545. Thus, DVU1545 has physiologies like MapA but domains more similar to LapF. Recently, Karbelkar et al. compared the predicted biofilm regulatory components to those of *P. fluorescens* (15). Like the soluble, periplasmic protease, LapG, the protease encoded by DVU1019 in *D. vulgaris* Hildenborough cleaves DVU1012 at a di-alanine motif (106AA107). However, a transmembrane (TM) helix in the protein DVU1019 is proposed to anchor DVU1019 to the inner membrane and is essential for function. LapD binds c-di-GMP by a catalytically inactive c-di-GMP phosphodiesterase domain (EAL) (26). Under high c-di-GMP concentrations LapD binds LapG and sequesters it from cleaving LapA. Under low c-di-GMP concentrations, LapG is released and cleaves LapA, releasing it from the cell surface and allowing the cell to detach from the surface (27). Karbelkar et al. showed that a similar protein, encoded by DVU1020, contains a catalytically inactive phosphodiesterase domain, HD-GYP, instead of EAL domain, but has a high affinity for c-di-GMP and modulates DVU1019 protease activity, similar to the LapD-LapG complex. How this complexation is impacted by DVU1019 being bound to the inner membrane remains unknown. For simplicity, Karbelkar et al. referred to these proteins as DvhA-F analogous to LapA-F. Our current study and these studies have revealed differences in these biofilm systems. Given these differences, we propose that these proteins be named for their function in the biofilm formation system (Bfs). We propose the following naming: BfsA for the adhesin encoded by DVU1012; BfsEBC for DVU1013, DVU1017, and DVU1018 encoding the type I secretion system components TolC, ATP transporter, and membrane fusion protein; BfsG for the protease encoded by DVU1019, BfsD for the effector protein encoded by DVU1020; and BfsF for the adhesin DVU1545. These subunits maintain the connection between the Bfs and Lap systems but acknowledge the functional differences between these systems and will facilitate further comparative studies.

## MATERIALS AND METHODS

### Strains, media, and growth conditions

The construction and storage of the parental strain (Δ*upp*; JWT700; used as wild type in this study) and the in-frame, markerless biofilm adhesin mutants ΔDVU1012 (JWT705), ΔDVU1545 (JWT706), and double-deletion mutant (ΔDVU1012 ΔDVU1545; JWT709) used in this study have been described previously (10). The bacterium *D. vulgaris* DP4 was provided by Judy Wall and grown under the same conditions as the *D. vulgaris* Hildenborough strains. Cultures were prepared in MOYLS4; LS4D with 60 mM sodium lactate and 50 mM sodium sulfate (60:50); or LS4D with 20 mM sodium lactate, 16.6 mM sodium sulfate, and 106 mM sodium chloride (20:16.6), as described previously and indicated where relevant below (10). All incubations were done at 30°C unless otherwise indicated. Starter cultures were prepared by inoculating 1 mL of a frozen stock into 10 mL of anoxic MOYLS4 within a sealed Balch-type 18 × 150 mm^2^ tube with a nitrogen headspace. These were incubated until the cultures reached an optical density at 600 nm (OD_600_) of approximately 0.8 (mid- to late-log phase) measured with a Genesys 30 Visual Spectrophotometer (Thermo Scientific, Waltham, MA, USA). Tubes for culturing biofilms under batch and static conditions were prepared by placing a halved borosilicate glass slide (Fisher Scientific, Hampton, NH, USA) sitting obliquely in 10 mL of 60:50 LS4D within an anoxic Balch tube and sterilized by autoclaving. Cultures were inoculated at a 1:50 dilution of the starter culture and incubated for 24 h. At the end of each experiment, to check for aerobic contamination of cultures, 10 µL of each culture was inoculated onto LC + glucose agar plates, prepared as described previously (10), and incubated at the same temperature as the experimental conditions (either 30°C or 23°C).

### Biofilm formation under continuous culture and shear conditions

To determine differences in biofilm protein composition, strains WT, ΔDVU1012, and ΔDVU1545 were cultivated within the CDC bioreactors containing glass slides (Biosurface Technologies, Bozeman, MT, USA) as done previously (10). Reactors containing mild steel coupons (1018 carbon steel; Biosurface Technologies) washed with acetone followed by 95% (vol/vol) ethanol to remove residue from manufacturing were set up in a similar manner but were filled with 1× phosphate-buffered saline (28) before sterilization by autoclave to prevent abiotic corrosion and sparged with nitrogen gas before aseptically draining the buffer solution. Briefly, starter cultures of WT, ΔDVU1012, and ΔDVU1545 were used to inoculate 20:16.6 LS4D media within the CDC bioreactors and incubated at 30°C. Reactor headspaces were continuously sparged at 100 mL/min with nitrogen gas passed through a sterile gas filter. Once the cultures within the bioreactors reached an OD_600_ of ~0.7, media were flowed into each reactor at 0.7 mL/min. Biofilm and planktonic samples were taken daily for five consecutive days. At 72 h, biofilm samples were taken in triplicate for proteomic analyses. All biofilm and planktonic samples were pelleted via centrifugation (17,000 × *g*, 2 min) and stored at −20°C. Planktonic samples from 120 h were used for genomic DNA extraction using the Wizard Genomic DNA Purification Kit, (Promega, Madison, WI, USA). Genotypic confirmation of each strain was done using PCR as previously described (10). Protein from biofilm pellets was quantified via the Bradford Assay (29).

### Biofilm formation under batch culture and static conditions

Strains of DvH were grown from freezer stock until reaching the late log phase of growth (OD_600_ ~ 0.8). Strains were then sub-cultured in a 1:50 dilution into anoxic Balch tubes containing 60:50 LS4D medium either with or without a halved glass slide in triplicate and incubated at 30°C under static conditions. OD_600_ measurements were taken over time to track planktonic growth over time. After 24 h of growth, biofilm samples were taken from each strain as reported previously (10). Biofilm samples were then stored at −20°C until quantified via the microassay protocol of the Bradford Assay (29, 30).

### Capillary flow cell imaging

Square glass capillary tubes (Friedrich & Dimmock, Millville, NJ, USA) measuring 1 × 1 × 100 mm^3^ with an average wall thickness of 0.17 mm were cleaned with 95% EtOH and connected to polypropylene-derived tubing (Avantor, Radnor Township, PA, USA). Capillary and Masterflex L/S 13 Norprene tubing (Avantor Inc., Radnor, PA, USA) were sterilized via autoclave (121°C, 30 min), and then aseptically connected. The capillaries were then placed within a 3D-printed holder and then secured onto the mechanical stage of a DMi8 epifluorescence microscope (Leica, Deerfield, IL, USA).

Once cultures showed signs of active growth in 60:50 LS4D, samples of WT, ΔDVU1012, ΔDVU1545, and ΔDVU1012 ΔDVU1545 were used for direct cell counts in a Neubauer counting chamber (Levy Ultra Plane; Clay-Adams Co., New York, NY, USA) and the cultures were diluted to 1 × 10^6^ cells/mL with 60:50 LS4D. About 30 mL of each culture was then aseptically transferred to the capillary flow cell system and then recirculated through the tubing and capillaries at 300 µL/min for 10 min to homogenize the cultures within the tubing. A Masterflex L/S Digital Drive with a standard pump head for L/S 13 tubing was used to recirculate the cultures at 0.002 mL/min within the flow cell system. During this time, five positions were chosen at random on each capillary tube for imaging. After 10 min, flow was reduced to 2 µL/min for image acquisition. Microscopy images were taken through an HC PL FL L 63×/0.70 CORR PH2 objective lens (NCI, Brooklyn Park, MN, USA) and a K5 microscope camera (Thomas Scientific, Chadds Ford Township, PA, USA). Phase contrast images of each inner capillary surface were taken every 10 min for 48 h at each position, with autofocus correction occurring every 1 h. Image series were then saved for processing and analysis. Image processing and analysis were performed using Leica Application Suite X v.3.7.4.23463 (LAS X) to determine attachment events for cells and cell aggregates, as well as to measure cell and cell aggregate sizes. Object size and location data were used to track particles across each image series with LAS X.

### Peptide sample preparation

Proteins were extracted from biofilm samples as done previously with modifications noted here (31, 32). Briefly, biofilm pellets were resuspended in 1 mL lysis buffer (10 mM Tris-HCl pH 8.0, 1% wt/vol SDS, 0.1 M dithiothreitol (DTT)) and incubated at 95°C for 30 min. Biofilm samples were then sonicated on ice (20% amplitude, 30-s intervals, five intervals) using a Branson 450 digital sonifier with a 102C converter (Branson Electronic Corp., St. Louis, MO, USA). Lysed biofilm samples were then centrifuged at 14,000 × *g* and 4°C for 10 min. Supernatants were transferred to new tubes before adding trichloroacetic acid to a final concentration of 25% (vol/vol) and stored at 4°C overnight to precipitate solubilized proteins. Samples were then pelleted via centrifugation (20,800 × *g*, 10 min), the supernatant discarded, and the pellets washed three times with chilled (~4°C) acetone. Protein pellets were solubilized in guanidine buffer (6 M guanidine HCl, 10 mM DTT in Tris CaCl_2_ buffer [50 mM Tris, 10 mM CaCl_2_, pH adjusted to 7.6 with HCl]) at 60°C, 1,500 rpm for 1 h before mixing with UA buffer (8 M urea in 0.1 M Tris-HCl pH 8.5) in a 1:3 ratio and concentrated on a 30 kDA filter (MilliporeSigma, Burlington, MA, USA) via centrifugation (14,000 × *g*, 15 min). Columns were washed with UA buffer, IAA buffer (50 mM iodoacetamide in UA buffer), and then ABC buffer (50 mM NH_4_HCO_3_) via centrifugation as done in the previous step. Concentrated protein extracts were then digested with 3 µL trypsin according to the manufacturer’s instruction (Promega, Madison, WI, USA) at 37°C overnight. Digested protein samples were then eluted with ABC buffer and desalted using the Pierce C-18 Spin Columns according to the manufacturer’s instructions (Thermo Scientific, Waltham, MA, USA). Peptide samples were then immediately quantified using the Pierce Quantitative Colorimetric Peptide Assay (ThermoScientific, Waltham, MA, USA). Peptides were then frozen at −80°C, then lyophilized at −50°C and 0.015 mBar for 6 h. Lyophilized peptides were then sent to the IDeA National Resource for Quantitative Proteomics (Little Rock, AR, USA) for proteomic analyses.

### Proteomic analysis

Tryptic peptides were separated from other peptides with reverse phase XSelect CSH C18 2.5 µm resin (Waters, USA) on an in-line 150 × 0.075 mm^2^ column using an UltiMate 3000 RSLCnano system (ThermoScientific, Waltham, MA, USA). Separated peptides were then eluted using a 90-min gradient from 98:2 to 65:35 buffer A:B ratio (Buffer A: 0.1% formic acid, 0.5% acetonitrile; Buffer B: 0.1% formic acid, 99.9% acetonitrile). Eluted peptides were ionized by electrospray at 2.4 kV, followed by mass spectrometric analysis on an Orbitrap Eclipse Tribrid mass spectrometer (ThermoScientific, Waltham, MA, USA). Mass spectrometry (MS) data were acquired using the FTMS analyzer in profile mode at a resolution of 120,000 over a range of 375 to 1,200 *m/z*. Following HCD activation, MS/MS data were acquired using the ion trap analyzer in centroid mode and normal mass range with a normalized collision energy of 30%. Proteins were identified using Sipros v1.1 (33) and quantified using ProRata v1.1 (34). Briefly, Sipros Ensemble was used to search MS spectra against the proteins of *D. vulgaris* Hildenborough acquired from https://microbesonline.org. Raw search results were filtered to achieve a 1% false discovery rate at the peptide level, which was estimated using the target-decoy approach. Peptide identifications were assigned to protein or protein groups in accordance with the parsimonious rule. Protein quantification was achieved through intensity-based label-free analysis using ProRata. Protein abundances were quantified by the total peak height of all quantified peptides from a protein, normalized against the average total of all data sets. Relative fold change analysis of identified protein intensities was done using Excel and methods derived from Aguilan et al. (35). Briefly, protein intensities were normalized before determining fold change differences between WT and biofilm adhesin mutants. An *F*-test was performed to determine whether the normalized protein intensities were homoscedastic or heteroscedastic, and then a Student’s *t* test with independent samples was performed either using the homoscedastic or heteroscedastic selection. The cutoff for significant fold changes was set to either positive or negative 2, and *P* < 0.05. Localization of proteins from *D. vulgaris* Hildenborough was predicted using PSORTb v.3.0.3 (36). Functional categories of proteins were determined using the Cluster of Orthologous Groups (COGs) database (37).

## Data Availability

The raw mass spectrometry data from the proteomics analysis have been deposited in the EMBL-EBI Proteomics Identifications Database (PRIDE) (38) with the data set identifier PXD056366.

## SUPPLEMENTAL MATERIAL

Figures S1 and S2 jb.00379-24-s0001.pdf Fig. S1: Biofilm formation on steel. Fig. S2: Biofilm formation by *D. vulgaris* DP4.

Table S1 jb.00379-24-s0002.xlsx Protein intensities, transformations, and normalizations for biofilm proteomic analyses.

ASM does not own the copyrights to Supplemental Material that may be linked to, or accessed through, an article. The authors have granted ASM a non-exclusive, world-wide license to publish the Supplemental Material files. Please contact the corresponding author directly for reuse.
