# Supplementary material for: Absence of biofilm adhesin proteins changes surface attachment and cell strategy for Desulfovibrio vulgaris Hildenborough
Source: J Bacteriol. 2024 Dec 31;207(1):e00379-24. doi: 10.1128/jb.00379-24 (PMC11784015; doi:10.1128/jb.00379-24)
Supplement: Figures S1 and S2 — Fig. S1: Biofilm formation on steel. Fig. S2: Biofilm formation by D. vulgaris DP4. [file jb.00379-24-s0001.pdf]

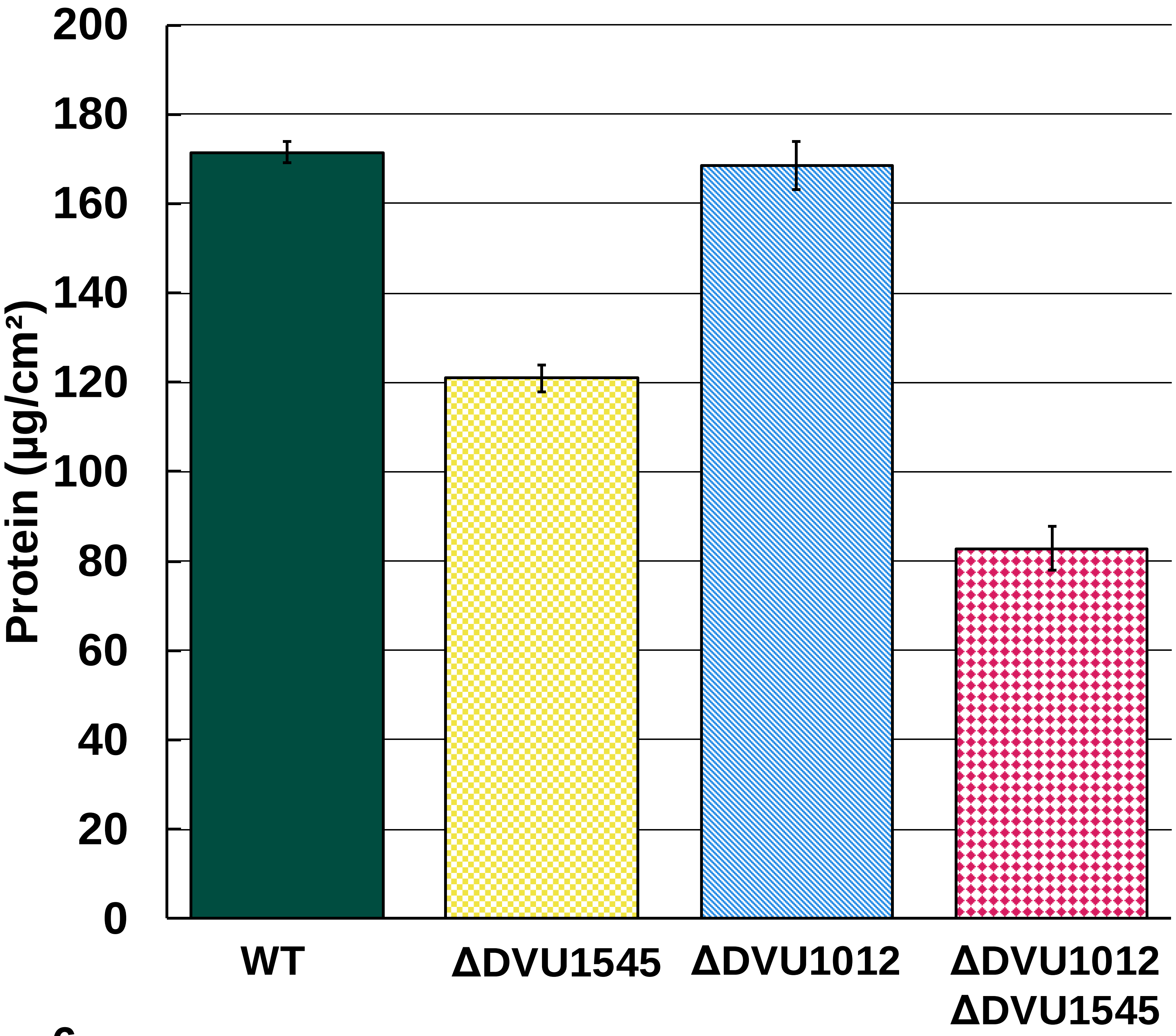

Figure S1. Biofilm growth for WT and the biofilm adhesin mutants on mild steel surfaces. Biofilms from CDC bioreactors were quantified as protein per square centimeter after 72 h. Error bars indicate standard deviation across technical replicates (n=3).

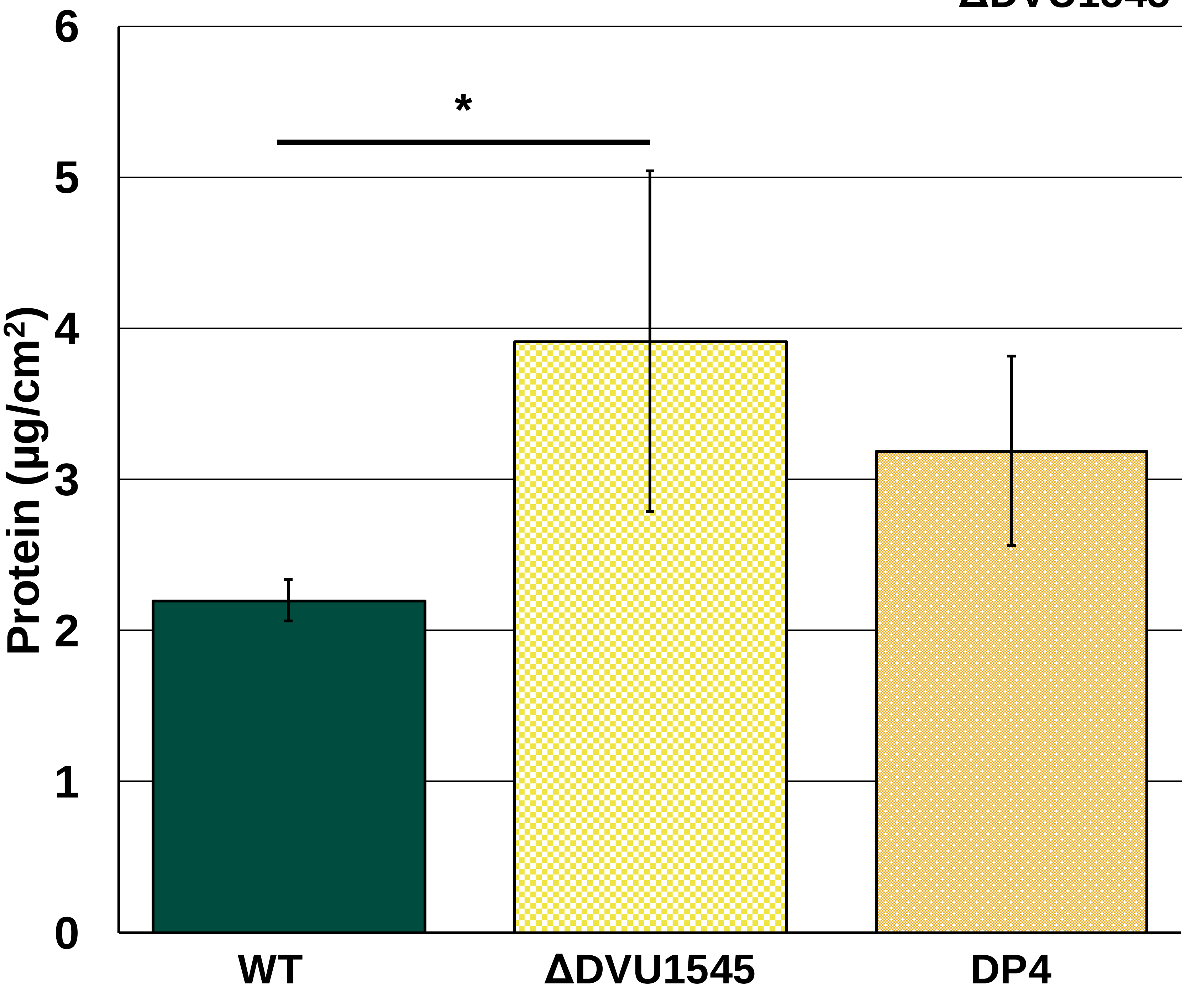

Figure S2. Early biofilm growth under static conditions for *D. vulgaris* strains Hildenborough (WT and ΔDVU1545) and DP4. Error bars indicate standard deviation across replicates (n=3). Line and asterisk above samples indicate statistically significant differences as calculated with a one-way ANOVA and Tukey-Kramer post hoc test ($p < 0.05$).
